# The Medicinal Uses of Ergot, Other Than as a Parturifacient

**Published:** 1862-11

**Authors:** M. O. Heydock


ARTICLE XXXVII.

### THE MEDICINAL USES OF ERGOT, OTHER THAN AS A PARTURIFACIENT.

By M. O. HEYDOCK, M.D.

Read before the Chicago Medical Society.

The cure of a disease is brought about by the removal of its proximate cause; and this is effected either by the *vis medicatrix naturæ*, or conjointly with the assistance of art. The careful observer learns, as far as is possible, in what way nature accomplishes the result unaided, and discovers that under certain circumstances, it is through a greater activity of the skin, the intestinal canal, or the renal organs. Having noted that nature spontaneously removes many derangements of the animal economy, his next step is to learn what drugs will accomplish like results when nature fails. The accumulated experience of medical men for ages has furnished us with many medicines, mineral and vegetable, which we may rely upon as positive agents. Croton oil is a drastic cathartic, given internally, and an irritant applied externally; the Spanish fly blisters the surface; and sulphate of zinc or ipecacuanha causes emesis.

The physician, should he so elect, may stop at this point, satisfied with the accomplished fact, not caring how the results are brought about, or in other words, the *modus operandi* of this drug, he goes on, skilful perhaps in diagnosis, and so far as the evacuant treatment is of benefit, using his remedies with a fair degree of success. But in that class of cases for instance, where the question arises whether the extreme vessels are dilated or contracted, and whether a drug has the power to cause or remove either of these morbid conditions, he finds himself at fault.

I use this illustration because the subject I propose discussing in this evening's paper has, with several others, been ably presented to the profession by BROWN SEQUARD, in a series of articles upon some forms of paralysis. I know that there are those who think that ergot was designed by Providence for parturient women alone, and consequently that we should be satisfied that in so doing it has accomplished its mission; it is an absurdity, say they, to bring it forward as a remedy in paralysis; we have in strychnine the only agent we want, and if this will not cure our patient, nothing will.

I cannot subscribe to these opinions, believing, as I do, that there is no department of our professional study which to-day repays labor and research like the unfolding of the hidden treasures of our materia medica.

Each year is fruitful of some discovery, which explains and makes clear to the student the reason *why* certain results are brought about with a good degree of certainty by certain remedies; some great practical truth is developed which—engrafted upon sound views of physiology and pathology—enables him to apply a remedy in cases where accident and good fortune might never have inclined him.

I shall relate to you opinions gleaned from various authors and writers, in reference to the use of ergot, aside from its action as a parturifacient, many of which go to support SEQUARD'S theory, which you are aware is that many cases of paralysis are attended with, or caused by, hyperæmia, or congestion of the vessels of the spinal cord, while in others the opposite condition exists; and that the remedy indicated in one class of cases fails in or aggravates the other, and his acknowledged success in the treatment of the grave lesions entitles his opinion to our confidence and respect. He claims that ergot, belladonna, and mercury cause contraction of the extreme vessels of the cord; while strychnine and brucia cause congestion and fulness of those vessels; and he uses them as he thinks this or that condition exists.

I shall not seek to support his theory, but by citing extracts in reference to the different uses to which the ergot has been put, leave each to decide whether he can reconcile the action with the hypothesis.

He believes that belladonna acts with greater energy upon those of the brain, while ergot is preferable where we wish to reach those of the spinal cord. In large doses, this drug is said to produce a sense of pain and weight in the head, giddiness, dilitation of the pupils, delirium, and stupor; and, according to Wood & Bache, a reduction in the frequency of the pulse.

Its long continued use has, we know, caused terrible and devastating epidemics in France, and different parts of the continent of Europe; death occurring, in many instances, which could be traced directly to the use of bread contaminated with the presence of this degenerate grain.

Prominent among the symptoms, to which its use gave rise, were dry gangrene, great disturbance of the nervous system, attended with convulsion, typhus fever, and kindred disorders.

Wood asks if it may not have the power of producing contraction of the capillaries in general, or of interfering in some other way with the circulation of the blood in these vessels, as by the exertion of a direct sedative or paralizing influence on them, as in this way we may explain the dry gangrene, resulting from its use as well as its influence in restraining hemorrhage.

Dr. C. L. Mitchell, of Kings County, New York, read a paper upon its use in spermatorrhœa, congestion, and irritation of the genital organs of the male.

He says that it was first used in diarrhœa in 1840, and about one year afterwards, in various hemorrhages. He now used it in dysmenorrhœa, which he supposed to arise from congestion of the uterus and its appendages, and with success.

Its use was attended with gratifying results in cases of spermatorrhœa.

The second case treated of this nature, was that of a physician, who was a confirmed opium eater, and a great sufferer from seminal emissions, the combined influence of which had reduced him to a condition not much removed from dementia; great benefit was derived from its exhibition within seven days.

In a case of great irritation about the neck of the bladder, ergot and camphor were given; and though it was of several days' duration, and a severe one, relief followed the first dose.

A writer in the *Medical Gazette*, of 1848, says that he finds that ergot, taken as a snuff, will remove the excessive dilitation of the pupil, arising from belladonna, and thinks that it may be successful in dilated pupil from other causes.

Dr. ROUSSELL, of St. Bartholemew's Hospital, speaks highly of its use in pulmonary hemorrhage, after various remedies had been tried in vain, the first dose controlling it.

Dr. PAGAN, in the *Medical Gazette*, of 1842, records three cases of paraplegiu treated by it successfully; one of which was the result of saturnine poisoning.

Mr. WRIGHT, in an article upon the oil of ergot, speaks of its use in capillary hemorrhage, bleeding from leech bites, the extraction of a tooth, and diarrhœa.

Numberless cases are reported in *Braithwaite*, and other journals, of its use in retention of urine. One case where it had been complete, it yielded after a few days' use of ergot, in doses ranging from 10 grains to half a drachm. One girl, who had long suffered from this disorder, but who was under treatment for amenorrhœa, was entirely relieved by six doses.

Dr. MITCHELL, to whose paper I previously referred, reports a case where the catheter had been required for a space of three months, as being entirely cured by this treatment. Its value in these cases cannot be denied.

Dr. BARKER, of New York, says that he seldom has occasion to use the catheter in the retention following labor; and since I noticed his suggestion, I resort to its early use, and in many cases have derived much benefit from its administration.

Dr. ALLIER, in the *London Lancet*, of 1848, laid down the following conclusions:—

I. Ergot restores to the bladder its contractility, impaired by immoderate distension of its coats, and has acted in this manner when all other means have failed.

II. Paralysis of the bladder, arising from cerebral hemorrhage, has promptly given way under its use; but it has no effect in paralysis of the limbs, resulting from apoplexy.

III. It shortens the duration of vesical paralysis, failing once in fourteen times, which is about the proportion of approved remedies.

IV. Its effects are rather evanescent, and it should, therefore, be frequently repeated; the dose may be 60 or 70 grs. a-day, and it should be gradually reduced after apparent recovery.

V. He sums up by saying that we have in the systematic use of ergot a remedy capable of shortening vesical paralysis, which after a longer or shorter time, *may* yield to other means; but which is often looked upon as incurable, and often producing that sad and disgusting infirmity—incontinence of urine.

I have now presented such facts and observations as I have found recorded; and I think that very many of them support the theory advanced. If, in the crucible of experience, this position is sustained, we have a remedy of great value, and one of which the profession will not be slow to avail itself. If, on the other hand, it prove like many which have proceeded it—beautiful in theory but inert and powerless for good in practice—we shall, at any rate, have the consolation of feeling that we have not labored in vain, for a negative result in one direction is not necessarily barren of influence upon events and discoveries which follow after.

